# Know when to fold ‘em: Polycomb complexes in oncogenic 3D genome regulation

**DOI:** 10.3389/fcell.2022.986319

**Published:** 2022-08-29

**Authors:** Emma J. Doyle, Lluis Morey, Eric Conway

**Affiliations:** ^1^ School of Biomolecular and Biomedical Science, Conway Institute, University College Dublin, Dublin, Ireland; ^2^ Sylvester Comprehensive Cancer Centre, Miami, FL, United States; ^3^ Department of Human Genetics, Biomedical Research Building, University of Miami Miller School of Medicine, Miami, FL, United States; ^4^ Smurfit Institute of Genetics, Trinity College Dublin, Dublin, Ireland

**Keywords:** Polycomb, H2AK119ub1, H3K27me3, chromatin architecture, cancer, compaction, transcription

## Abstract

Chromatin is spatially and temporally regulated through a series of orchestrated processes resulting in the formation of 3D chromatin structures such as topologically associating domains (TADs), loops and Polycomb Bodies. These structures are closely linked to transcriptional regulation, with loss of control of these processes a frequent feature of cancer and developmental syndromes. One such oncogenic disruption of the 3D genome is through recurrent dysregulation of Polycomb Group Complex (PcG) functions either through genetic mutations, amplification or deletion of genes that encode for PcG proteins. PcG complexes are evolutionarily conserved epigenetic complexes. They are key for early development and are essential transcriptional repressors. PcG complexes include PRC1, PRC2 and PR-DUB which are responsible for the control of the histone modifications H2AK119ub1 and H3K27me3. The spatial distribution of the complexes within the nuclear environment, and their associated modifications have profound effects on the regulation of gene transcription and the 3D genome. Nevertheless, how PcG complexes regulate 3D chromatin organization is still poorly understood. Here we glean insights into the role of PcG complexes in 3D genome regulation and compaction, how these processes go awry during tumorigenesis and the therapeutic implications that result from our insights into these mechanisms.

## Introduction

Cell fate regulation is a critical developmental process, governed carefully by transcriptional control. This results in the ability of a given cell to activate and repress specific gene sets, culminating in a great diversity of cell types and transcriptional phenotypes. While it has long been appreciated that the 3D structure of chromatin is critical to the determination of cell fate and maintenance of cellular identity, technological advances of the last decade have facilitated a renaissance in the molecular dissection of genome architecture (Jerkovic and Cavalli, 2021). Moreover, there is a burgeoning understanding of the protein complexes and molecular mechanisms responsible for regulating chromatin architecture. These layers of topological genome regulation require precise temporal and spatial coordination to control gene expression.

Foremost among those protein complexes that control genome architecture and transcription are the Polycomb group complexes (PcG). These are a family of highly conserved histone modifying multi-subunit complexes that are essential for development (Lagger et al., 2002; Schuettengruber et al., 2017). Moreover, PcG dysfunction has been implicated as a driving force in several human diseases and syndromes (Conway et al., 2015; Deevy and Bracken, 2019; Tamburri et al., 2022).

The ever-expanding repertoire of genomics techniques to study the regulation of genome architecture has allowed a deep molecular understanding of the modes of PcG-mediated compaction (Jerkovic and Cavalli, 2021). In this review, we will summarize the contribution of the PcG complexes and their respective catalytic activities to the control of genome topology and how this is perturbed in cancer. We speculate on the future direction of research into the role of PcG in chromatin structure and the therapeutic opportunities that a mechanistic understanding of PcG-dysregulated cancers can provide.

### Catalytic activities of Polycomb group complexes

The PcG machinery can be divided into three major complexes: Polycomb Repressive Complex 1 and 2 (PRC1 and PRC2) and Polycomb Repressive Deubiquitinase complex (PR-DUB). Traditionally, PcG complexes are associated with maintenance of gene repression, mainly *via* histone-modifying activities. PRC2 catalyses methylation at lysine 27 of histone H3 (H3K27me1/2/3) through its catalytic core of EZH1/2, SUZ12, EED, and RBBP4/7 (Cao et al., 2002; Ferrari et al., 2014). PRC1 deposits a ubiquitin group on lysine 119 of histone H2A (H2AK119ub1) *via* the E3-ligases RING1A/B which heterodimerize with one of the six PcG RING finger domain (PCGF1–6) paralogs (Wang et al., 2004a; de Napoles et al., 2004; Gao et al., 2012). In mammals, PRC1 can be further subdivided into two main complexes, namely canonical or variant PRC1 (cPRC1 and vPRC1), though all PRC1 complexes contain either RING1A or RING1B (Gao et al., 2012). H2AK119ub1 is erased through the activity of deubiquitinating enzymes such as BAP1, USP16 and MYSM1 (Joo et al., 2007; Zhu et al., 2007; Scheuermann et al., 2010; Yang et al., 2014; Rong et al., 2022). BAP1 forms part of the PR-DUB complex, which consists of a catalytic core of BAP1 and one of three ASXL paralogs (ASXL1-3). PR-DUB has a particularly prominent role in oncogenesis (Carbone et al., 2013).

**FIGURE 1:**
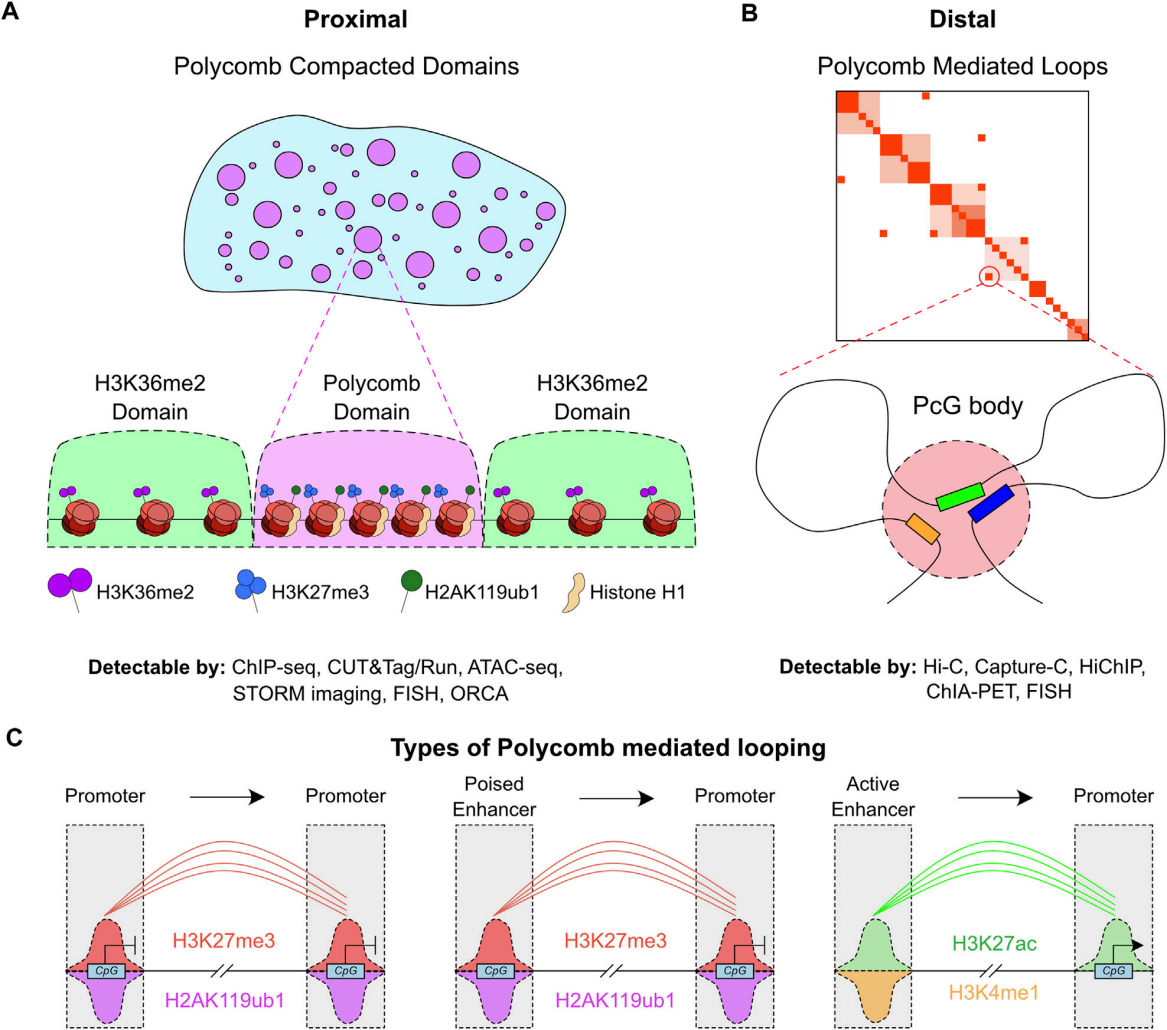
Divergent mechanisms of chromatin structure regulation by PcG. **(A)** Illustration of nuclear (blue) clusters containing Polycomb domains (purple). These proximal domains are occupied by H3K27me3, H2AK119ub1 and Histone H1, while boundary domains are enriched in H3K36me2 (green). Techniques that can be used to measure Polycomb domains and their associated compaction and histone modifications are listed underneath the graphic. **(B)** Illustration of distal Polycomb mediated loops, with example chromatin capture contact map, which involve multiple regions that are distal in 3D-space contacting each other in stable Polycomb bodies. Techniques that can be used to measure Polycomb mediated loops are listed underneath the graphic. **(C)** Cartoon of the different genomic features that undergo Polycomb mediated looping, and the histone modifications associated with these features.

These PcG complexes are responsible for two distinct forms of chromatin structural features (Figures 1A,B); either proximal compaction or distal looping. The proximal form, ‘PcG compacted domains’ can perhaps be better considered as compaction of the linear 2D genome (Figure 1A), through careful ordering of neighboring nucleosomes in regions enriched by PcG modifications. This coordinated process, accompanied by accumulation of the linker histone H1, promotes local compaction. These proximally compacted sites correlate well with ‘B compartments’ which are inactive regions that tend to be in close proximity to other inactive regions (Jerkovic and Cavalli, 2021; Willcockson et al., 2021; Yusufova et al., 2021). Such domains are interspersed by ‘A compartments’ that are enriched in active genes or broad domains enriched in the H3K36me2 modification that is associated with more permissive or open chromatin (Figure 1A).

The distal form of ‘PcG mediated loops’, more commonly termed PcG bodies, involves mid/long-range loops from one PcG bound region to another. These can be observed at distances of up to 100 MB (Figure 1B) (Schoenfelder et al., 2015; Kraft et al., 2022; Ngan et al., 2020). This type of compaction is perhaps better studied than the proximal form, but its precise contribution to transcriptional regulation is still an open question. A number of forms of these PcG-mediated loops have been reported, with the general mechanism reporting loops between PcG bound promoters (Figure 1C) (Ngan et al., 2020). However, studies to date have been limited to certain cell-types which may prevent a complete understanding of the dynamic role of PcG-mediated loops in transcription and development. Similar loops have been discovered between ‘poised’ or PcG marked enhancers with transcriptionally repressed genes (Figure 1C) (Rada-Iglesias et al., 2011; Pachano et al., 2021).

**FIGURE 2:**
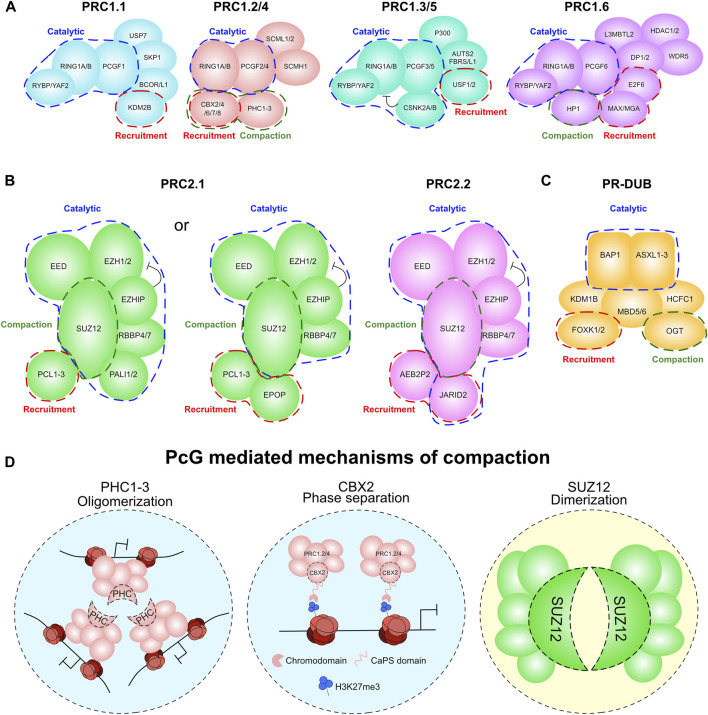
PcG complexes and their contribution to compaction. **(A)** The variable compositions of the PRC1 complex. Compaction related subunits are highlighted in green. Catalytic related subunits are highlighted in blue. Recruitment related subunits are highlighted in red. **(B)** The variable compositions of the PRC2 complex. Compaction related subunits are highlighted in green. Catalytic related subunits are highlighted in blue. Recruitment related subunits are highlighted in red. **(C)** The composition of the PR-DUB complex. Compaction related subunits are highlighted in green. Catalytic related subunits are highlighted in blue. Recruitment related subunits are highlighted in red. **(D)** Model of the compaction mechanism for PcG proteins PHC1-3, CBX2 and SUZ12.

There is huge diversity in the proteomic composition of the PcG complexes in mammals (Gao et al., 2012; Morey et al., 2012; Hauri et al., 2016; Gahan et al., 2020; Kang et al., 2022) with specific activities and biological functions. For instance, we and others have shown that cPRC1 and vPRC1 complexes have distinct biological functions (Morey et al., 2013; Rose et al., 2016; Fursova et al., 2019; Scelfo et al., 2019). Notably, their specific gene targets, recruitment mechanisms, and enzymatic activities are context dependent, and they exert specialized functions during the early stages of development. These differential activities are generally driven by the specific combinatorial assembly of PcG accessory proteins in each sub-complex. The molecular function of many PcG subunits have been well defined, with three general means through which a subunit can contribute to the function of a PcG complex: chromatin recruitment, catalytic regulation, and compaction/structural functions (Figures 2A–C). Here we will focus on the catalytic and compaction roles of PcG subunits as they are the direct contributors to PcG-mediated 3D genome architecture. The mechanisms of PcG recruitment to chromatin have been reviewed in depth elsewhere (Glancy et al., 2021; Tamburri et al., 2022).

While the catalytic products of these complexes are clearly correlated with transcriptional repression of nearby genes, the precise contribution of each histone modification in maintaining or initiating repression is still incompletely understood (Pengelly et al., 2013; Pengelly et al., 2015; Blackledge et al., 2020; Tamburri et al., 2020; Dobrinic et al., 2021). Catalytic inactivation of the PRC1 complex in embryonic stem cells (ESC) rapidly depletes H2AK119ub1 levels in the cell, resulting in concurrent upregulation of PRC1 target genes (Blackledge et al., 2020; Tamburri et al., 2020; Dobrinic et al., 2021). This highlights a clear direct contribution to transcriptional repression. However, this process is apparently independent of PRC2 complex activity as H3K27me3 levels are largely maintained. In fact, knockout or deletion of PRC2 fails to induce transcriptional upregulation in ESC (Tamburri et al., 2020; Dobrinic et al., 2021), despite clear importance in transcriptional repression in adult stem cells, differentiation and development (Pengelly et al., 2013; Chiacchiera et al., 2016; Sankar et al., 2022). Together this suggests that the role of H2AK119ub1 is at least partially independent of PRC2. While there are many reported sensors of H2AK119ub1, it is as yet unclear which, if any, of these contribute to the role of this modification in repression (Tamburri et al., 2022).

#### Dynamic functions of PcG

The PcG complex cores act as the scaffold upon which auxiliary subunits bind to confer their distinct functions, including proteins that are involved in recruitment such as CBXs for cPRC1, KDM2B or MAX/MGA for vPRC1, PCL1-3 or JARID2 for PRC2 and FOXK1/2 for PR-DUB (Figures 2A–C) (Farcas et al., 2012; Endoh et al., 2017; Healy et al., 2019; Kolovos et al., 2020). Importantly, while these targeting proteins are required for maintaining stable tethering of complexes to target sites such as promoters or enhancers, emerging evidence shows that the function of PcG complexes is not limited to those long-term residences. H2AK119ub1, H3K27me2 and H3K27me3, although specifically enriched at these tethered sites, are also broadly distributed throughout the genome (Ferrari et al., 2014; Lee et al., 2015; Fursova et al., 2019). This is in stark contrast to the occupancy of the PcG complexes from ChIP studies, suggesting a low/temporary residency of these complexes at chromatin. Indeed, live-cell imaging studies have allowed the dissection of the dynamics of PRC1 and PRC2 complexes (Youmans et al., 2018; Huseyin and Klose, 2021). Remarkably, only 10%–20% of PRC1 complexes and ∼20% of PRC2 complexes are bound to chromatin at any given time in ESC. This supports a ‘hit and run’ model in which the PcG complexes are diffusing throughout the nucleus generating sparse ‘blanket’ levels of their modifications, and when they reach a site for which they have a higher affinity (such as CpG islands) they can become stably tethered, but the majority of PcG complexes are in a catalytically active state of flux.

While the precise function of these intergenic, diffuse histone modifications is yet to be fully understood, work to date suggests that they can contribute to the maintenance of proximal PcG compacted domains. Whether this is through a direct role of the modifications, or their established antagonisms with other epigenetic modifications such as H3K36me2, DNA methylation or RNA molecules remains to be fully elucidated.

#### Intra-complex rheostats of catalytic potency in PcG

The biochemical diversity of PcG provides these complexes with tools to allosterically modulate the activities of their catalytic cores. These rheostats within the PcG complexes fine tune histone modifying activity to maintain an equilibrium of H2AK119ub1 and H3K27me3 at specific regions in the genome. PRC2 is a prime example of this, with a positive regulator of allosteric activity within each of its two subcomplexes, PALI1/2 within PRC2.1 and JARID2 within PRC2.2 (Figure 2B) (Sanulli et al., 2015; Conway et al., 2018; Zhang et al., 2021a). Each of these are methylated themselves by EZH2 (at residue K1241 for PALI1 and K116 for JARID2), these modifications are recognised by the aromatic cage of EED which promotes allosteric activity of the PRC2 complex. Genetic approaches aimed at perturbing the allosteric activity of PRC2 complexes will shed light into how PRC2 modulates its own enzymatic activity and will determine the phenotypic implications of these intra-complex rheostats in controlling cell fate determination and cellular homeostasis.

PRC2 can also associate with an inhibitory subunit, EZHIP (Figure 2B). EZHIP is mainly expressed in gonads, where it inhibits PRC2 activity through a domain mimicking the H3K27M oncohistone (Jain et al., 2019; Piunti et al., 2019; Ragazzini et al., 2019). This mechanism blocks PRC2 activity *in trans* and prevents its spreading from nucleation sites at CpG islands (Jain et al., 2020b). It is not yet known whether other mammalian proteins contain the same domain of EZHIP responsible for PRC2 inhibition.

PRC1 shares similar positive and negative regulators of catalytic activity. RYBP and YAF2 subunits, which can be a part of any of the vPRC1 complexes, promote catalytic activity, though the structural basis for this is not yet known (Figure 2A) (Morey et al., 2013; Rose et al., 2016; Zhao et al., 2020). This possibly utilises the H2AK119ub1 binding affinity for the paralogous RYBP/YAF2 subunits in order to facilitate spreading of H2AK119ub1 from existing modified sites (Kalb et al., 2014; Cooper et al., 2016). Moreover, chromatin compaction dependent on the linker histone H1 plays a critical role in the stable propagation of H2AK119ub1 by RYBP/YAF2–PRC1 (Zhao et al., 2020). The negative regulators of PRC1 exist specifically within the PCGF3 and PCGF5-containing PRC1.3/5 complexes (Figure 2A). These are the two most catalytically active forms of the complex *in vitro* and contribute to the bulk of H2AK119ub1 in ESC (Taherbhoy et al., 2015; Fursova et al., 2019; Conway et al., 2021). However, the PRC1.3/5 complexes contain the CSNK2A and CSNK2B kinases which can phosphorylate RING1B at S168 resulting in diminished catalytic function *in vitro* (Gao et al., 2014). It is unknown whether this inhibitory phosphorylation occurs in a physiological setting, indeed its relevance to PRC1 biology also remains to be fully elucidated.

### Contribution of Polycomb group complexes to compaction

Each of the three PcG complexes can serve structural roles that facilitate chromatin compaction and structural maintenance such as through PcG bodies. PcG bodies are foci in the nuclei containing H2AK119ub1 and H3K27me3 (Isono et al., 2013). Imaging and high-throughput chromosome conformation capture experiments revealed that PcG bodies contain PcG bound loci that interact in 3D space, even though they can be separated by very large distances across the genome (Boyle et al., 2020). The key player in PcG body formation is PRC1 (Francis et al., 2004). This is supported by PRC1 knockout studies showing loss of local and distal compaction/looping, and decompacted nuclear architecture in its absence (Schoenfelder et al., 2015; Boyle et al., 2020; Rhodes et al., 2020).

More specifically, cPRC1 containing either PCGF2/4, CBX (CBX2/4/6/7/8) and the Polyhomeotic (PHC1-3) subunits (Gao et al., 2012; Morey et al., 2012) are recruited to chromatin through the affinity of the chromodomain within each CBX subunit for the PRC2-mediated modification H3K27me3 (Fischle et al., 2003; Wang L. et al., 2004). The role of cPRC1 in distal regulation of compaction and looping is generally considered to be through their PHC subunits. These are essential for normal embryonic development, with a dose-dependent function in maintaining homeotic gene repression (Isono et al., 2005). PHC1-3 contribute to cPRC1 mediated repression through hetero-oligomerisation via their Sterile Alpha Motif (SAM) domain (Figure 2D). This results in compaction of cPRC1 marked regions into distinct PcG body speckles (Isono et al., 2013; Boettiger et al., 2016; Kundu et al., 2017). This PHC1-3 mediated aggregation controls looping of PcG-bound loci to each other, most strikingly at *HOX* genes which are particularly prominent PcG target loci. Most significantly, mutation of the PHC2 SAM domain prevents cPRC1 aggregation and induces homeotic transformations in developing mice, showing that this role of PHC1-3 is critical to PRC1 function (Isono et al., 2013).

The CBX subunits of cPRC1 are primarily involved in cPRC1 recruitment to chromatin. However, the CBX2 subunit has unique properties in proximal chromatin compaction that seemingly cause phase separation of PcG target loci (Figure 2D) (Plys et al., 2019; Tatavosian et al., 2019). In Drosophila, this function is controlled by the Psc (PCGF ortholog) subunit, but in mammals is unique to CBX2 (Grau et al., 2011). This function of CBX2 is conferred by an intrinsically disordered Compaction and Phase Separation (CaPS) domain. This domain is enriched in basic residues that are essential for the capacity of CBX2 to phase separate (Plys et al., 2019; Tatavosian et al., 2019). Intriguingly, this domain is also critical for appropriate repression of PcG target genes during embryogenesis, with mutations in these basic residues causing homeotic transformations (Lau et al., 2017). This CaPS domain also confers an impaired ability to remove PcG, suggesting that loci in these condensates have altered dynamics of transcriptional regulation (Jaensch et al., 2021). This is consistent with the observed immobility of CBX2 on chromatin relative to the other CBX proteins, and indeed the rest of cPRC1 in live cell imaging studies (Zhen et al., 2014). While the function of this ability of CBX2 to form condensates is still incompletely understood, it does afford CBX2-PRC1 the capacity to condense DNA and chromatin into discrete particles (Tatavosian et al., 2019).

Therefore, the PHC1-3 and CBX2 subunits confer distinct modes through which cPRC1 can regulate chromatin compaction. Despite this, the precise contribution of cPRC1 to PcG mediated repression remains a matter of debate, relative to its more catalytically active variant (vPRC1) forms containing PCGF1/3/5/6 (Taherbhoy et al., 2015; Fursova et al., 2019). In fact, acute perturbation of cPRC1 via *PCGF2* and *PCGF4* double knockout in ESC fails to induce major gene expression changes (Fursova et al., 2019). The same is clear in adult stem cells such as skin stem cells, where double loss of *PCGF2* and *PCGF4* does not recapitulate the *RING1A/B* knockout phenotype (Cohen et al., 2018). Notwithstanding these mild phenotypes in acute systems, constitutive mutants of cPRC1 result in homeotic transformations and defects in differentiation (Akasaka et al., 1996; Jacobs et al., 1999; Akasaka et al., 2001; Morey et al., 2015). Together this may suggest that cPRC1 is more important during long-term cell fate transitions and is dispensable for transcriptional repression in static model systems. Therefore, short-term PcG repression is maintained by factors other than compaction but establishing *de novo* PcG repressive domains may require cPRC1 mediated compaction.

PR-DUB contributes to PcG-mediated repression through the catalytic activity of BAP1 by removing H2AK119ub1 (Scheuermann et al., 2010; Sahtoe et al., 2016). However, it can also contribute to the function of cPRC1 complexes in chromatin looping. Drosophila lacking the glycosyltransferase enzyme *Ogt* exhibit failure to repress PcG targets akin to *Ph* mutants (the Drosophila PHC paralog) (Gambetta and Muller, 2014). Biochemical analysis attributed this phenotype to a requirement for glycosylation of a serine/threonine rich domain on Ph to prevent non-productive aggregation of Ph or PHC proteins (Gambetta and Muller, 2014). Therefore, OGT contributes to PcG repression through prevention of aberrant PHC hetero-oligomerisation, maintaining PcG compaction in a precisely regulated manner (Figure 2D). In mammals, OGT is a subunit of the PR-DUB complex (Hauri et al., 2016; Kloet et al., 2016). However, while Ogt is clearly important for PcG-mediated repression, it remains to be seen whether this is a PR-DUB related function, and what physiological function the glycosylation of PHC has on PcG body formation in mammals.

The role of PRC2 in directly controlling compaction is less well defined than PRC1. Although, emerging biochemical data suggests two distinct modes through which PRC2 can contribute to chromatin compaction. These are through i) SUZ12-mediated dimerisation and ii) direct nucleosomal compaction by EZH1 (Figure 2D). While it has been known for some time that PRC2 is capable of dimerisation (Davidovich et al., 2014), the structural basis for this has recently been elucidated. SUZ12 can undergo ‘domain switching’ between its C2 domain and the WDB1 domain on another molecule of SUZ12 resulting in dimerisation of two core PRC2 complexes (Chen et al., 2020; Grau et al., 2021). Moreover, loss of this dimerisation capacity leads to defects in H3K27me3 deposition at PcG target genes and compaction of nucleosomal arrays.

EZH1 can promote compaction of nucleosomal arrays independently of catalytic activity and interestingly, its compaction function is stronger than that of its more catalytically active paralog EZH2 (Margueron et al., 2008; Lee et al., 2018; Grau et al., 2021). Structural studies have revealed that this function of EZH1 can be attributed to a divergent basic patch in the MSL2 loop of EZH1. This domain promotes nucleosome binding and indeed compaction *in vitro*. This divergent role of EZH1 in compaction is strikingly similar to the divergent evolution of PRC1 subcomplexes with cPRC1 having weaker enzymatic potency than vPRC1 but instead maintaining functions in chromatin compaction (Blackledge et al., 2014; Gahan et al., 2020), much like the differences in the compaction and catalytic activities of EZH1 and EZH2 (Margueron et al., 2008; Lee et al., 2018; Lavarone et al., 2019; Grau et al., 2021).

The biological contributions of the OGT, SUZ12 and EZH1-mediated compaction mechanisms to PcG biology has yet to be fully uncovered as the studies to date have generally been *in vitro*. Discrete mutagenesis experiments will allow the dissection of their contributions to PcG-mediated compaction and transcriptional repression.

### Polycomb body disruption in cancer

An ever-expanding concept in cancer genetics over the last decade is the recurrent disruption of transcriptional and epigenetic machinery (Vogelstein et al., 2013). The components of the PcG complexes are a strong example of this, with mutations in their subunits driving many cancers in a context-dependent fashion (Conway et al., 2015). The effect of these mutations on PcG-mediated 3D-structure has been under explored to date. This is particularly urgent as fully elucidating these mechanisms may facilitate the discovery of epigenetic targeted therapies to restore chromatin architecture to a healthy state.

**FIGURE 3:**
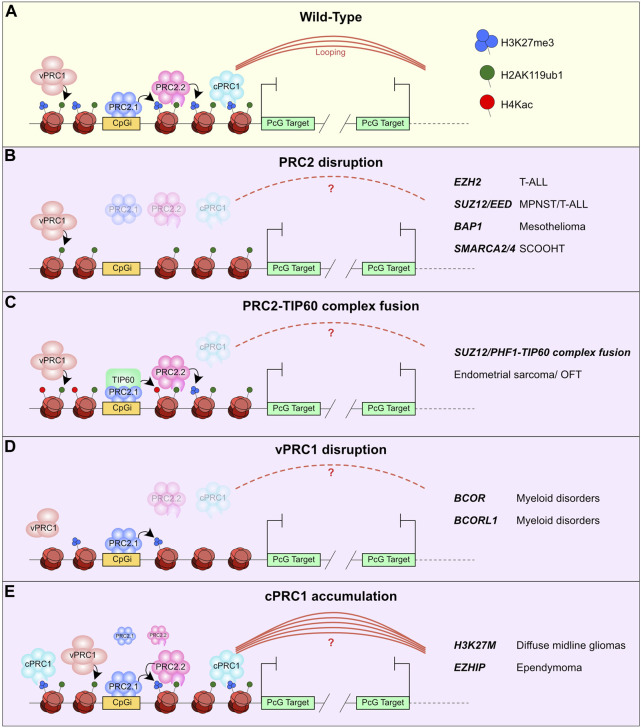
Malformation of PcG bodies in cancer. **(A)** Schematic of recruitment model for PRC1 and PRC2 complexes. i) vPRC1 is recruited by transcription factors or CpGi readers and catalyses H2AK119ub1. ii) PRC2.2 reads H2AK119ub1 and PRC2.1 is recruited to CpGi, these complexes catalyse H3K27me3. iii) cPRC1 reads H3K27me3 and promotes Polycomb mediated looping to other cPRC1 marked regions. **(B–E)** Illustration of how different mutations of PcG proteins in diverse cancer types affects the PcG recruitment pathway and in turn cPRC1 mediated looping. This includes **(B)** PRC2 disruption, **(C)** PRC2-TIP60 complex fusion, **(D)** vPRC1 disruption, **(E)** and cPRC1 accumulation. The genes mutated and the cancer they are associated with are listed on the right.

PcG bodies that control distal looping of PcG bound elements are primarily regulated through the cPRC1 complex. Disruption of these avenues of looping or aggregation can occur through a myriad of mutations affecting the PcG recruitment pathway. A simplified version of this pathway is that: i) vPRC1 is recruited to chromatin through DNA binding at CpG islands or transcription factor binding sites (Farcas et al., 2012; Wu et al., 2013; Endoh et al., 2017; Scelfo et al., 2019). ii) These complexes catalyse H2AK119ub1 which promotes PRC2 recruitment either directly through PRC2.2 sampling H2AK119ub1 or PRC2.1 affinity for CpG islands (Cooper et al., 2016; Choi et al., 2017; Healy et al., 2019; Hojfeldt et al., 2019; Blackledge et al., 2020; Tamburri et al., 2020). iii) These PRC2 subcomplexes in turn catalyse H3K27me3, thus prompting recruitment of CBX-containing cPRC1 and causing aggregation of these sites into PcG bodies (Figure 3A) (Fischle et al., 2003; Min et al., 2003; Blackledge and Klose, 2021).

Loss-of-function (LOF) mutations of *EZH2* occur in several cancers including T-cell Acute Lymphoblastic Leukemia (T-ALL) and Acute Myeloid Leukemia (AML) (Ntziachristos et al., 2012; Zhang et al., 2012; Dohner et al., 2017; Liu et al., 2017). The *SUZ12* and *EED* genes also feature LOF mutations in T-ALL and are particularly frequent in Malignant Peripheral Nerve Sheath tumours (MPNST) (Lee et al., 2014; Zhang et al., 2014). These PRC2 LOF mutant cancers have reduced H3K27me3 (Ntziachristos et al., 2012; Lee et al., 2014; Wassef et al., 2019) and therefore may be deficient in cPRC1 mediated looping, as loss of H3K27me3 can prevent cPRC1 recruitment and aggregation (Figure 3B).

A similar disruption of PcG distal looping may occur through other chromatin modifier-mutated cancers such as *BAP1* in mesothelioma, uveal melanoma, and renal clear cell carcinomas (Figure 3B) (Testa et al., 2011; Carbone et al., 2013). Recent studies have shown that PR-DUB and the catalytic function of BAP1 are required for maintenance of PRC2 target gene occupancy and PcG-mediated repression in ESC and cancer cells (Scheuermann et al., 2010; Abdel-Wahab et al., 2012; Conway et al., 2021; Fursova et al., 2021). Diffuse accumulation of H2AK119ub1 in the absence of *BAP1* causes titration of PRC2 and cPRC1 from their target sites (Conway et al., 2021). Although *BAP1* loss causes widespread compaction, the specific effect of *BAP1* loss on PcG bodies remains to be investigated. Supporting the idea that *BAP1* mutant cancers actually confers loss of PcG repression rather than the previously assumed hyperactivation of PRC1 and PRC2 activities, is the lack of response of *BAP1* mutant mesothelioma patients to the EZH2 inhibitor Tazemetostat (Zauderer et al., 2022).

The PcG complex genes *SUZ12*, *PHF1*, EZHIP and *BCOR* undergo chromosomal translocations resulting in fusion proteins in endometrial stromal sarcoma and ossifying fibromyoxoid tumours (OFT) (Hrzenjak, 2016; Sudarshan et al., 2022). Intriguingly, their fusion protein partners can vary but are consistently part of the TIP60 (NuA4) complex family. The most common of these fusions being *JAZF1-SUZ12* fusions, but these PcG genes have also been reported to be translocated with other TIP60 complex subunits (Sudarshan et al., 2022). TIP60 is a histone acetyltransferase for histone H4 and is primarily found at sites of active transcription. These fusions have a profound effect on the histone modification landscape at PcG target genes with a reduction in H3K27me3 at target genes and gain in H4Kac (Sudarshan et al., 2022; Tavares et al., 2022). These alterations are likely caused by eviction of accessory subunits from the PcG complexes such as loss of EPOP, PALI1 and JARID2 (Chen et al., 2018; Piunti et al., 2019; Tavares et al., 2022). This biochemical disruption of the PRC2 complex disturbs PcG complex redistribution and gene repression during ESC differentiation. The reductions in H3K27me3 at PcG targets make it likely that PcG body formation is also disrupted with potential gains in active enhancer-promoter looping mediated by increased acetylation (Figure 3C), such as the observed gains in intra-TAD acetylation at the *HOXD* locus (Sudarshan et al., 2022).

Disruption of PcG bodies is not limited to cancers with mutations in PcG family members. In fact, contrary to canonical models of PcG-BAF complex antagonism (Kadoch et al., 2017), loss of SMARCA2/4 causes displacement of cPRC1 and PRC2 complexes from their targets and decompaction of PcG target loci including HOX gene clusters (Weber et al., 2021). This is intriguing as mutation of *SMARCA2* and *SMARCA4* occurs in several cancer types such as small cell carcinoma of the ovary hyper-calcaemic Type (SCCOHT) (Pan et al., 2019). This new data suggests that BAF mutant cancers may also reduce PcG target occupancy and therefore impair its looping activity (Figure 3B). The mechanism for this has not been fully elucidated but is reminiscent of the displacement of cPRC1 and PRC2 in the *BAP1* mutant context, which causes an imbalance in H3K27me3 and H2AK119ub1 that titrate the complexes away from their target sites.

Several components involved in catalysing/erasing/reading H2AK119ub1 are recurrently mutated in myeloid disorders such as AML, including *ASXL1*, *DNMT3A*, *BCOR* and *BCORL1* (Dohner et al., 2017). BCOR and BCORL1 are critical components of the PCGF1-containing vPRC1 complexes that catalyse promoter proximal H2AK119ub1 (Farcas et al., 2012; Wu et al., 2013). The *BCOR* and *BCORL1* nonsense and frameshift mutations occurring in AML patients result in C-terminally truncated proteins lacking the PUFD domain. This causes an uncoupling of KDM2B and BCOR/L1 from the rest of the complex, thus impairing vPRC1 recruitment and its catalytic activity at target loci (Schaefer et al., 2022). The subsequent reductions in PRC2 binding are in keeping with the established recruitment model and suggest that cPRC1 driven looping may be indirectly impaired by these mutations due to reductions in H3K27me3 (Figure 3D).

There are also several oncogenic events that can cause gain- or change-of-function to PcG complex activities. These include the *H3K27M* oncohistone mutation that occurs in diffuse midline gliomas, or expression of the gonad specific PRC2 subunit *EZHIP* in ependymoma (Sturm et al., 2012; Wu et al., 2012; Lewis et al., 2013; Pajtler et al., 2018). These events share a mechanistic impact on PcG function as they can broadly inhibit the catalytic activity of the PRC2 complex (Lewis PW 2013; Mohammad et al., 2017; Jain et al., 2019; Ragazzini et al., 2019), therefore they were initially thought to act as LOF mutations. Precise epigenomic mapping of PcG activities in these contexts has instead revealed that *H3K27M* mutation or *EZHIP* expression in fact allows maintenance of H3K27me3 at discrete PcG target loci, including tumour suppressor genes, throughout the genome, but there is a subsequent failure to spread H3K27me3 beyond these nucleating sites (Mohammad et al., 2017; Jain et al., 2020b; Brien et al., 2021). The consequence of this is increased PRC2 and cPRC1 binding at target loci (Brien et al., 2021). Therefore, a likely scenario is that these alterations have a hyper-compacting influence on PcG body formation, driven by high levels of cPRC1, preventing activation of differentiation stimuli under PcG repression (Figure 3E).

Taken together there are a myriad of mechanisms through which PcG-mediated looping can be directly or indirectly disrupted in cancer. Whether these compaction changes are themselves involved in oncogenesis or driving aberrant gene expression changes remains largely unexplored. However, exciting new technologies and more cost-efficient methodologies (Jerkovic and Cavalli, 2021) makes investigating these mechanisms a high priority for the future of both PcG and cancer fields.

### Polycomb at distal regulatory elements to support gene transcription

**FIGURE 4:**
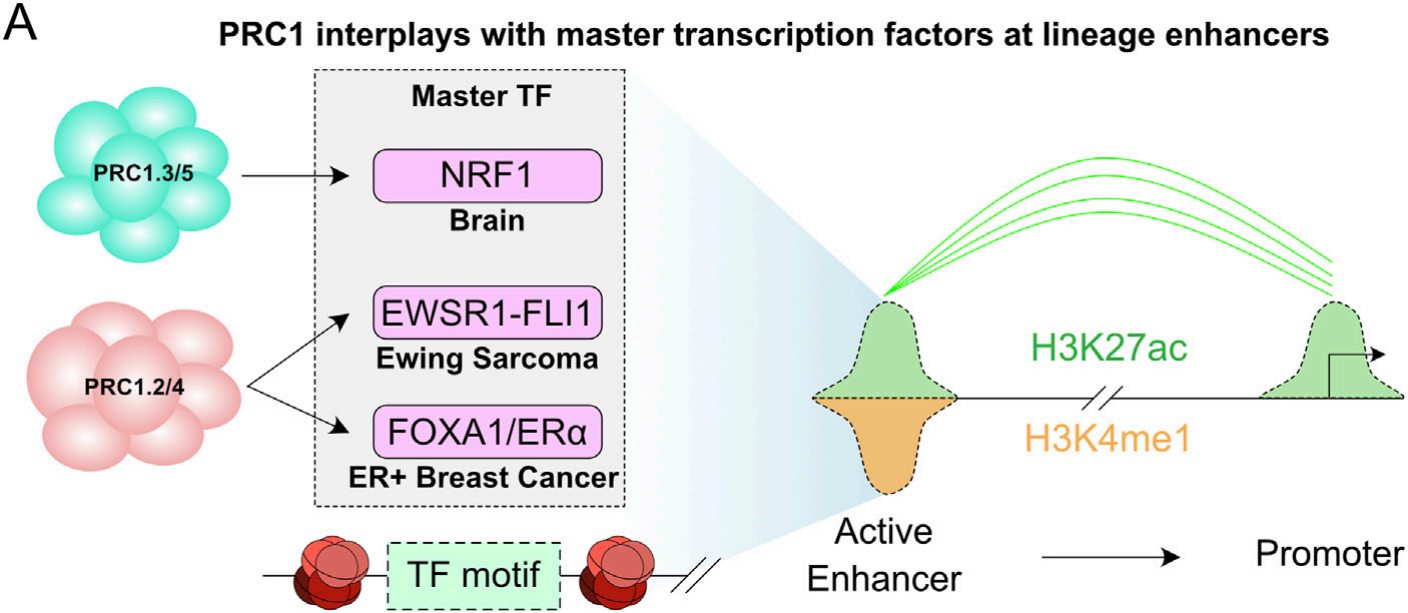
Polycomb at distal regulatory elements to support gene transcription. **(A)** Illustration of the interplay between PRC1 and master transcription factors showing their requirement for enhancer activity in different disease and tissue contexts.

While PcG complexes have been classically associated with gene repression, especially in pluripotent cells, recent evidence suggests that formation of PcG domains is also required for bringing enhancers within close proximity of gene promoters that are rapidly induced by differentiation stimuli (Figure 1C). This proposes a model in which PcG complexes are also required for rapid induction of gene transcription (Cruz-Molina et al., 2017; Ngan et al., 2020; Pachano et al., 2021). Similarly, an elegant study by Koseki and colleagues showed that RING1B is required for both repression and activation of *Meis2* during early midbrain mouse development by regulating the promoter and enhancer interaction in a spatial and temporal manner (Kondo et al., 2014). Moreover, during neuronal differentiation, RING1B associates with active enhancers and enhancer-promoter interactions (Loubiere et al., 2020) and can associate with AUTS2 and p300 to drive neuronal differentiation by the transcription factor nuclear respiratory factor 1 (NRF1) (Figure 4A) (Gao et al., 2014; Liu et al., 2021).

Association of PRC1 with gene transcription has also been linked to adult stem cell differentiation. Work on epidermal stem cells suggested that PRC1 complexes promote the expression of skin regulatory genes. Although PRC1 still exerts its repression function in epidermal stem cells, a large cluster of PRC1 occupied sites contain histone modifications associated with active enhancers and promoters (H3K27ac, H3K4me3 and H3K4me1) (Cohen et al., 2018). Therefore, PRC1 may be required to promote transcription of key skin regulatory genes but it might also be associated with active enhancers and 3D chromatin organization to maintain cellular homeostasis. Similarly, RING1B is required for gene activation in resting B cells, and analysis of RING1B binding shows occupancy of RING1B at promoters and distal sites, suggesting a role of RING1B in regulating enhancer-promoter interactions to promote gene transcription (Frangini et al., 2013).

During tumorigenesis it has been shown that PRC1 complexes are recruited to both transcriptionally repressed and active genes, as well as at active enhancers. Interestingly, PRC1 is co-recruited with oncogenic transcription factors (TFs) to active enhancers in estrogen receptor positive (ER+), triple-negative breast cancer (TNBC), leukemia, hepatocellular carcinoma cell lines and Ewing sarcomas (Chan et al., 2018; Sánchez-Molina et al., 2020). This suggests that PRC1 is a master epigenetic regulator that associates with master TFs to drive tumorigenesis. For instance, in Ewing sarcomas expressing the oncogenic transcription factor EWSR1-FLI1, RING1B colocalizes with EWSR1-FLI1 at active enhancers to regulate its recruitment to chromatin and promote transcription (Figure 4A) (Sánchez-Molina et al., 2020). Similarly, in breast cancer cells RING1B is not the main H2AK119ub1 E3-ligase, and RING1A seems to be more enzymatically active (Chan et al., 2018). In ER+ breast cancer, the steroid hormone estrogen recruits PRC1 to ERα target genes. RING1B depletion demonstrated that it is required for full ERα recruitment, estrogen-mediated transcription, R-loop formation, and enhancer-promoter interactions upon estrogen stimulation (Figure 4A) (Zhang et al., 2020; Zhang et al., 2021b). Although the exact PRC1-mediated mechanism that promotes gene transcription in breast cancer cells is not fully understood, similarly to PRC2 (Long et al., 2020), RNA is required for PRC1 stabilization at chromatin upon estrogen induction (Zhang et al., 2021b). Whether other steroid hormones, such androgens and progesterone, regulate PRC1 recruitment to activate oncogenic pathways in other cancer types is not known.

### Polycomb domain boundaries in stem cells and cancer

While H3K27me3 and H2AK119ub1 are particularly enriched at repressed promoters (in proximity to sites of stable PcG binding and CpG islands) a large proportion of these modifications are distributed at low “blanket”-like levels throughout the intergenic genome (Bracken et al., 2006; Ferrari et al., 2014; Lee et al., 2015; Fursova et al., 2019). These diffuse levels of H3K27me3 and H2AK119ub1 maintain “PcG domains” and often co-occur with high levels of H3K27me2, another catalytic product of PRC2 (Ferrari et al., 2014). It is thought that these diffuse modifications are catalysed by dynamic untethered PRC1 or PRC2 that are in a constant state of flux in the nucleus (Youmans et al., 2018; Huseyin and Klose, 2021). Another hypothesis is that these modifications can be generated shortly following DNA replication as PRC1 and PRC2 complexes have been observed in close proximity to the replication fork machinery (Hansen et al., 2008; Alabert et al., 2014; Piunti et al., 2014). Functional effectors for these diffuse or ‘blanket’ modifications are not well understood, with reader proteins such as CBX7 and JARID2 only stably binding to the highly enriched promoter proximal sites (Healy et al., 2019; Scelfo et al., 2019). Nevertheless, these PcG domains are precisely regulated through antagonistic interplay between PcG modifications and another epigenetic modification located at intergenic chromatin, H3K36me2. While the contribution of this careful balance of distal modifications to transcription is unknown, it has high relevance to chromatin structure and cancer as many of the proteins regulating this equilibrium of modifications are recurrently disrupted in cancer.

**FIGURE 5:**
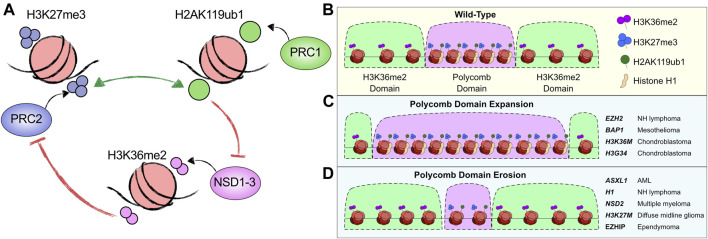
Dysregulated PcG domain formation in cancer. **(A)** Depiction of the protagonistic and antagonistic relationships between the histone modifications H3K27me3, H2AK119ub1 and H3K36me2 and the enzymatic complexes that catalyse them. **(B)** Schematic of Polycomb domains (B compartments) enriched in blanket levels of H3K27me3, H2AK119ub1 and occupied by Histone H1. These domains are boundaried by ‘A’ compartment related H3K36me2 enriched domains. **(C)** Illustration of how different mutations in PcG and other epigenetic machinery causes Polycomb domain expansion. The genes mutated and the cancer they are associated with are listed on the right. **(D)** Illustration of how different mutations in PcG and other epigenetic machinery causes Polycomb domain erosion. The genes mutated and the cancer they are associated with are listed on the right.

H3K36me2 is a modification deposited by the NSD1/2/3 histone methyltransferases (Deevy and Bracken, 2019). Structural studies have suggested a model in which NSD protein contacts with nucleosomes are sterically blocked by the presence of H2AK119ub1 (Figure 5A) (Li et al., 2021). Supporting this, H2AK119ub1 modified nucleosomes serve as a poor enzymatic substrate for NSD proteins compared to unmodified nucleosomes (Yuan et al., 2013; Li et al., 2021). A reciprocal antagonism is true for H3K36me2-modified nucleosomes which are an inefficient substrate for PRC2 complex activity (Yuan et al., 2011; Jain et al., 2020a). The presence of H3K36me2 on the histone tail reduces access of the SET domain of EZH2 to the H3K27 residue, as unmodified H3K36 is required to orient the H3 N-terminal tail correctly for optimal PRC2 activity (Finogenova et al., 2020). This reduction in efficiency of PRC2 in the presence of H3K36me2 explains the low correlation between H3K27me3 and H3K36me2 in the genome (Figure 5B), although H3K27me2 and H3K36me2 do coexist at intergenic sites (Streubel et al., 2018). This may be due to the rapid enzyme kinetics required for PRC2 catalysis of H3K27 di-methylation compared to long kinetics required for tri-methylation (Sneeringer et al., 2010; McCabe et al., 2012a). Accordingly, experiments that disrupt either PcG or NSD activities result in a switch in the balance of histone modification domains. For instance, in the absence of NSD1, H3K27me3 becomes predominant (Streubel et al., 2018), while in *BAP1* null cells, broad accumulation of H2AK119ub1 causes reductions in H3K36me2 (Conway et al., 2021).

A further layer of regulation to the PcG and H3K36me2 domains comes in the form of histone H1. The linker histone H1 binds to nucleosomes at the dyad and maintains ordered structure of the surrounding chromatin. This in turn has been shown to promote catalytic function of PRC1 as it facilitates spreading of PcG modifications across nucleosomes (Zhao et al., 2020). Accordingly, histone H1 loss causes invasion of PcG domains by H3K36me2 (Willcockson et al., 2021; Yusufova et al., 2021). The structural basis for this promotion of PRC1 catalytic activity has not yet been evaluated. However, histone H1 can stabilise the H2A C-terminal tail and promote its contact with DNA near the chromatosome dyad (Arimura et al., 2021; Zhou et al., 2021), which is in close proximity to the H2AK119 residue, perhaps implicating a direct role in promoting catalytic activity through structural manipulation of histone tails.

#### Invasive Polycomb domains

The balance of PcG domains has been directly implicated in oncogenesis of follicular lymphoma patients bearing *EZH2* gain-of-function mutations (e.g., Y641/A677) (Morin et al., 2010; McCabe et al., 2012a). These mutations alter the substrate preference for EZH2 from unmodified H3K27 to H3K27me1/2 facilitating an enzymatic progression to H3K27me2 by the wild-type EZH2 allele followed by rapid catalysis of H3K27me3 by the mutant EZH2 (McCabe et al., 2012a). These mutations were one of the earliest discovered PRC2 related dysregulations in cancer, and they feature global hyper-H3K27me3 (McCabe et al., 2012b). Importantly, the gains in H3K27me3 are not localised to promoter or CpG island regions but instead are primarily found in the ‘blanket’ intergenic regions (Souroullas et al., 2016; Donaldson-Collier et al., 2019). This coincides with increases in local compaction within TADs, inducing downregulation of germinal centre B-cell differentiation pathway genes such as *PRDM1* (Béguelin et al., 2016; Donaldson-Collier et al., 2019). Therefore, an altered PcG domain pathway causes a differentiation blockade through hyper-compaction of broad regions of chromatin (Figure 5C). Patients with these mutations are already benefiting from EZH2 inhibitors with early clinical results indicating promising response rates (Italiano et al., 2018).

Somatic missense mutations in genes encoding for canonical and variant histone H3 have been recently identified in a myriad of cancers (Sturm et al., 2012; Nacev et al., 2019). Mutations are often found in residues located in the histone N-terminal tail which plays fundamental roles in development, cancer, and gene regulation. Common mutations in genes encoding canonical histone H3 (H3.1) and variant histone H3 (H3.3, encoded by *H3F3A* and *H3F3B*) include lysine 36 to methionine (H3K36M) found in chondroblastomas, lysine 27 to methionine (H3K27M) found in pediatric gliomas, and glycine 34 to tryptophan/leucine (H3G34W/L) mutations found in giant cell tumors of the bone (Lewis et al., 2013; Lu et al., 2016; Jain et al., 2020a). Expression of H3K36M or H3G34W/L inhibits NSD proteins leading to global reduction of H3K36 methylation concomitantly with an accumulation of H3K27me3 at intergenic regions (Lu et al., 2016; Jain et al., 2020a). Notably, these oncohistones induce a similar phenotype to *EZH2* GOF (gain-of-function) mutations through removal or reduction in H3K36 methylation levels. These reductions in H3K36me2 cause widespread accumulation of PcG domains via diffuse spreading of H3K27me3 in turn promoting hyper-repression of distal regulatory elements (Figure 5C) (Jain et al., 2020a).

PcG domains are similarly altered upon *BAP1* LOF (loss-of-function) mutations and *EZH2* GOF mutations (Conway et al., 2021; Fursova et al., 2021). *BAP1* null or catalytic mutant cells exhibit broad increases in H2AK119ub1 and H3K27me3, due to the displacement of PcG complexes from promoters. This in turn enhances either their dynamism, their catalytic activity or perhaps both. Domains featuring gains in H3K27me3 in *BAP1* null contexts exhibit concurrent losses of H3K36me2 (Conway et al., 2021). Indeed, this coincides with global gains in compaction and nucleosomal reorganisation into packed clusters, and an increase in the levels of histone H1 on chromatin. Together with *H3K36M*, *H3G34W/L* and *EZH2* mutant cancers, these data highlight a parallel mechanism for PcG domain expansion/invasion and blockade of differentiation pathways in distinct cancer types (Figure 5C).

#### Polycomb domain erosion

The PR-DUB subunit ASXL1 is frequently mutated in myeloid malignancies such as AML (Dohner et al., 2017). Most *ASXL1* mutations are nonsense or frameshift mutations that result in a truncated protein lacking a C-terminal PHD domain (Tamburri et al., 2022). Unlike the LOF *BAP1* mutations observed in cancer, these truncating *ASXL1* mutations result in hyper-deubiquitination activity for PR-DUB complexes (Balasubramani et al., 2015). The mechanisms proposed for this increase in catalytic activity have been quite divergent with some reports suggesting that truncated ASXL1 either stabilises BAP1 protein levels (Bai et al., 2021; Wang et al., 2021) or loses interaction with the PR-DUB co-factors FOXK1/2 (Xia et al., 2021). FOXK1/2 are required for stably targeting PR-DUB to chromatin, but do not contribute to the catalytic activity of the complex (Kolovos et al., 2020). Together this may suggest that the ASXL1-truncated PR-DUB could have increased rates of dynamic activity at diffuse “blanket sites” or PcG domains. The effect of these mutations has yet to be fully established and whether hyper-DUB activity is restricted to PRC1 bound sites or broad PcG domains remains to be elucidated. If the latter, then it is possible that AML patients harbouring these ASXL1 truncations have restricted PcG domains with low levels of H2AK119ub1 and H3K27me3, potentially causing decompaction of chromatin into a naïve stem-like cell state (Figure 5D).

The *H3K27M* mutations in diffuse midline glioma (DMG) and *EZHIP* overexpression in ependymoma, while featuring increased levels of H3K27me3 at tumor suppressor genes such as *CDKN2A*, have broadly reduced levels of H3K27me3 and H3K27me2. This is caused by the inability to spread these modifications from PcG nucleation sites at promoters (Jain et al., 2020b). Concurrently, hyper-acetylation of H3K27 has been observed broadly in the absence of diffuse PcG activity (Krug et al., 2019; Brien et al., 2021). This results in the aberrant transcription of repetitive elements (Krug et al., 2019) and likely leads to a decompacted and stem-like chromatin state which is permissive to uncontrolled proliferation. Similarly, linker histone H1 encoding genes *H1-2*, *H1-3* and *H1-4* are recurrently mutated in follicular lymphoma. These mutations result in the degradation of PcG domains and invasion of these regions by H3K36me2 (Figure 5D) (Willcockson et al., 2021; Yusufova et al., 2021). This coincides with a switch from B to A compartments at these loci supporting their decompaction in the absence of PcG activity and histone H1 (Yusufova et al., 2021).

The *NSD2* gene exhibits recurrent GOF mutations in acute lymphocytic leukemia (Oyer et al., 2014; Swaroop et al., 2019). This *NSD2* E1099K mutation disrupts an autoinhibitory loop allowing enhanced catalytic activity (Sato et al., 2021). *NSD2* is also recurrently overexpressed in multiple myeloma through a t(4; 14) translocation that places the *NSD2* gene under the control of the *IgH* immunoglobulin promoter (Lhoumaud et al., 2019). Both events promote hyper-activity of NSD2, increasing H3K36me2 domains (Lhoumaud et al., 2019; Narang et al., 2022). This causes the erosion of PcG domains through inhibition of H3K27me3 and further coincides with large-scale changes in genome architecture (Figure 5D). This includes a general shift from B compartments (inactive) to A compartments (active), coinciding with those regions that gain H3K36me2 and lose H3K27me3 (Lhoumaud et al., 2019; Narang et al., 2022). These sites tend to become more transcriptionally active, and there is also an increase in enhancer activity. A further mechanism through which the balance of H3K36me2 and PcG domains may be affected is through *DNMT3A* mutations that occur in AML (Dohner et al., 2017). DNMT3A can read both H2AK119ub1 or H3K36me2 through its UIM and PWWP domains respectively (Weinberg et al., 2019; Weinberg et al., 2021; Gu et al., 2022). However, the myeloid mutations occur in its cytosine methyltransferase domain so it is not yet clear what effects these mutations can have on PcG domains. It seems likely that they cause a spreading of PcG domains due to the previously identified antagonism between DNA methylation and PcG.

Taken together, a careful balance of PcG and H3K36me2 domains appears to be important for the regulation of chromatin structure, plasticity, and differentiation potential. This is best highlighted by the fact that PRC2 and PR-DUB have both gain- and loss-of-function mutations in different cancer contexts (Morin et al., 2010; Ntziachristos et al., 2012; Balasubramani et al., 2015; Conway et al., 2021). Indeed, efficacy of EZH2 inhibitors in malignant rhabdoid tumours is dependent on the activity of NSD1, reinforcing the importance of this balance of Polycomb and H3K36me2 domains across different cancers (Drosos et al., 2022). The precise contribution of broad histone modification domains, largely devoid of epigenetic complex binding, remain unknown. Despite this, the many avenues through which these domains are recurrently disrupted in cancer highlights the importance of further investigation in this area in order to understand the oncogenic mechanisms and tailor appropriate epigenetic therapies to patients harbouring these mutations.

### Drugging the epigenome to restore PcG structural regulation

As epigenetic machineries are often deregulated or mutated in cancer (Chan and Morey, 2019), efforts in medicinal chemistry have led to the development of epidrugs to combat these dysregulated functions. Given the importance of the epigenome in promoting and maintaining human diseases, it is curious that to date only nine epidrugs are FDA-approved. These include inhibitors of EZH2, IDH, HDACs, and DNA hypomethylating agents. Nevertheless, during the last decade, dozens of small molecules that inhibit the enzymatic activity of epigenetic machineries have been developed. Notably, several epidrugs are undergoing clinical trials for treating solid and hematological malignancies. In this chapter, we will discuss recent progress on the development of small molecules that target specific PRC1 and PRC2 subunits (Table 1).

**TABLE 1:** PRC1 and PRC2 complex targeting compounds. Table detailing the published small molecule compounds that target PRC1 or PRC2 complex members and the mechanism of their targeting activity.

Target	Compound	Compound mechanism	Cancer types	References
***PRC2***				
EZH2	Tazemetostat (EPZ6438)	Catalytic Inhibitor	Non-Hodgkin’s lymphoma, malignant rhabdoid tumor	Italiano et al. (2018), Knutson et al. (2013)
	GSK126/GSK343	Catalytic Inhibitor	Non-Hodgkin’s lymphoma	McCabe et al. (2012b)
	EI1	Catalytic Inhibitor	Non-Hodgkin’s lymphoma	Qi et al. (2012)
	Astemizole/DC-PRC2in-01	EZH2-EED interaction Inhibitor	Non-Hodgkin’s lymphoma	Du et al. (2021)
	MS177	PROTAC	Mixed-lineage leukemia	Wang et al. (2022)
EZH1/2	UNC1999	Catalytic Inhibitor	Non- Hodgkin’s lymphoma	Konze et al. (2013)
EED	A-395	Allosteric Inhibitor	Non-Hodgkin’s lymphoma	He et al. (2017)
** **	EED226/EEDi-5285	Allosteric Inhibitor	Non-Hodgkin’s lymphoma	Qi et al. (2017), Rej et al. (2021)
** **	UNC6852	PROTAC	Non-Hodgkin’s lymphoma	Potjewyd et al. (2020)
***PRC1***				
RING1B	RB-3	Nucleosome-RING1B interaction inhibitor	Acute Myeloid Leukemia	Shukla et al. (2021)
	PRT4165	–	–	Ismail et al. (2013)
	GW-516	–	Prostate cancer	Su et al. (2019)
PCGF4 (BMI1)	PTC209	PCGF4 hyper-phosphorylation	Colorectal cancer	Kreso et al. (2014)
	PTC028	PCGF4 hyper-phosphorylation	Medulloblastoma, ovarian cancer	Bakhshinyan et al. (2019)
	PTC596	PCGF4 hyper-phosphorylation	Pancreatic ductal adenocarcinoma, acute myeloid leukemia	Eberle-Singh et al. (2019), Nishida et al. (2017)
CBX4/7	UNC3866	Chromodomain Inhibitor	Prostate cancer	Stuckey et al. (2016)
CBX7	UNC4976	Positive allosteric modulator DNA-binding	–	Lamb et al. (2019)
CBX8	SW2-110A	Chromodomain Inhibitor	Mixed-lineage leukemia	Wang et al. (2020)
	UNC7040	Positive allosteric modulator DNA-binding	Non-Hodgkin’s lymphoma	Suh et al. (2022)

#### Targeting PRC2 complexes

In homeostasis and non-malignant conditions, PRC2 suppresses expression of tumor suppressor genes and maintains cellular identity. EZH2 mutations and overexpression of core PRC2 subunits have been found in multiple solid cancers, including prostate and breast cancer, as well as in hematological malignancies (Comet et al., 2016; Chan and Morey, 2019). Therefore, PRC2 complexes and subunits are considered promising targets in cancer.

Notably, PRC2 can be targeted on multiples fronts and strategies should be tailored for each cancer type. For instance, initial studies were focused on targeting the enzymatic activity of EZH2. This led to the discovery of highly potent and selective compounds, including tazemetostat (McCabe et al., 2012b; Qi et al., 2012; Knutson et al., 2013; Konze et al., 2013; Italiano et al., 2018), which has been recently approved by the FDA. Alternatively, given the key role of EED in propagating H3K27me3 and maintaining PRC2 complex integrity, multiple small molecules targeting EED have been developed. EED directly interacts with EZH2, therefore Orkin and colleagues developed peptides that selectively disrupt the EED/EZH2 interaction, thereby reducing EZH2 protein levels as well as H3K27me3 in MLL-AF9-mediated leukemia (Kim et al., 2013). Also, small-molecule compounds that interfere with the EED/EZH2 interaction have been developed (e.g., astemizole and DC-PRC2in-01) and tested in PRC2-dependent follicular lymphomas (Du et al., 2021). Alternatively, recognition of H3K27me3 by EED suggested the possibility of developing allosteric inhibitors. Indeed, EED226 and A-395 bind to the H3K27me3 binding pocket of EED to allosterically inhibit PRC2 activity by inducing a conformational change of EED. Notably, both inhibitors reduced the growth of *EZH2* GOF mutant follicular lymphoma (He et al., 2017; Qi et al., 2017). Most importantly, these compounds are still effective in cancer cells that have acquired resistance to EZH2 inhibitors. Recently, analogs of EED226 (e.g., EEDi-5285) have been further developed and optimized with an enhanced binding affinity for EED and the ability to inhibit growth in mouse xenograft models of human follicular lymphoma (He et al., 2017; Rej et al., 2021).

Small molecules targeting EZH2, EED/EZH2 or EED/H3K27me3, although effective do not achieve complete tumor regression. Although an obvious consideration would be that a combination of PRC2 inhibitors with either other epidrugs or standard of care therapies would increase their efficacy, another possibility is that EZH2 has acquired non-canonical functions outside the context of PRC2. Indeed, it has been shown that in prostate cancer, breast cancer and in leukemia, EZH2 is also recruited to oncogenes to promote gene activation independently of its enzymatic activity (Lee et al., 2011; Xu et al., 2012; Chan and Morey, 2019; Wang et al., 2022). Therefore, compounds targeting PRC2 enzymatic activity or H3K27me3 propagation are unable to block aberrant EZH2 functions. This problem could be circumvented by the development of proteolysis targeting chimeras (PROTACs). PROTACs are bifunctional molecules containing a target protein ligand and an E3 ligase ligand. They induce selective degradation of their targeted proteins via the ubiquitin-proteasome system. Jin and Wang laboratories recently reported that the PROTAC MS177 achieved effective depletion of EZH2 in the context of both its canonical and non-canonical activities in MLL leukemias (Wang et al., 2022). Notably, they showed that tumor burden was more significantly reduced when mice were treated with MS177 instead of with UNC6852, a PROTAC that selectively targets EED (Potjewyd et al., 2020) and therefore the PRC2 activity but not the PRC2-independent activity of EZH2. Finally, given the specific roles of PRC2.1 and PRC2.2 complexes in development, cancer, and gene regulation, PROTACS targeting JARID2, and PCL proteins will certainly be developed in the near future.

#### Targeting PRC1 complexes

Although the majority of genes encoding for PRC1 subunits are not mutated in cancer, they are often deregulated (Chan et al., 2018; Chan and Morey, 2019). As mentioned above, dozens of PRC1 complexes can be assembled and simultaneously present in a given cell type. Thus, it is of great interest to develop small molecules and PROTACS that would either specifically target a discrete PRC1 complex or at least discriminate between cPRC1 and vPRC1 complexes, particularly in the context of cancers with high levels of H2AK119ub1, like *BAP1* mutant tumors. To date, small molecules targeting RING1B, PCGF4 and CBX proteins have been reported. Thus, compounds specifically targeting vPRC1 complexes have not yet been developed. We envision that PROTACs targeting RYBP, PCGF1, PCGF3, PCGF5 and PCGF6 will be reported in the coming years.

*RING1B inhibitors:* During the last decade, great efforts have been directed towards targeting RING1B, the core component of PRC1. To date, three small molecules targeting RING1B have been reported. Cierpicki and colleagues developed RB-3, the only available compound that directly interacts with RING1B (Shukla et al., 2021). RB-3 treatment leads to a reduction of H2AK119ub1 in AML cells. Notably, xenograft experiments showed that RB-3 was effective in reducing leukemias. Whether RB-3 is also effective in reducing tumor burden in cancers addicted to RING1B, such as breast and pancreatic cancers (Chen et al., 2014; Chan et al., 2018; Zhang Z. et al., 2021), remains to be investigated. PRT4165 and GW-516 were proposed to inhibit the E3-ligase activity of RING1B *in vitro*, although their mechanism of action is not known (Ismail et al., 2013; Su et al., 2019). While these compounds directly or indirectly regulate H2AK119ub1, high concentrations are required to inhibit RING1B’s activity, and therefore it is expected that more powerful analogs will be developed.

*BMI1 (PCGF4) inhibitors:* Several compounds that reduce BMI1 protein levels have been described. High-throughput screenings designed to identify compounds that reduced BMI1 transcript levels identified PTC209 (Kreso et al., 2014). Although PTC209 does not bind to BMI1, it reduces both BMI1 and H2AK119ub1 levels and inhibits the self-renewal of colorectal cancer-initiating cells, resulting in the abrogation of colorectal tumors. PTC028, an analog of PTC209, showed reduction in both primary medulloblastoma tumors and their metastatic sites, without neurotoxicity and led to increased survival in mice (Bakhshinyan et al., 2019). The growth of ovarian cancer cells, but not normal cells, and the tumor burden of an orthotopic ovarian cancer model are also reduced by PTC028 treatment. Mechanistically, BMI1 modulators inhibit the anaphase promoting complex APC/C leading to persistent cyclin-dependent kinase (CDK)1/2 activity and BMI1 hyper-phosphorylation (Dey et al., 2018). PTC596 also reduced BMI1 levels and H2AK119ub1 in pancreatic ductal adenocarcinoma (PDA) and AML (Nishida et al., 2017; Eberle-Singh et al., 2019), but recent studies challenged the specificity of this compound. Crystal structure studies revealed that PTC596 interacts with tubulin and inhibits microtubule polymerization (Jernigan et al., 2021). Moreover, elegant genetic studies using *Bmi1* null PDA cells, demonstrated that PTC596 induced mitotic arrest and had an antiproliferative effect independently of BMI1 (Eberle-Singh et al., 2019). Nevertheless, PTC596 is currently in clinical trials. A phase study in patients with advanced cancer (NCT02404480) has been completed. PTC596 was tolerable with gastrointestinal side effects. Currently, three phase 1b trials are ongoing in patients with high grade glioma (NCT03605550), advanced leiomyosarcoma (NCT03761095) and ovarian cancer (NCT03206645).

*CBX ligand inhibitors:* The ability of the Polycomb CBXs’ chromodomain to bind to H3K27me3 and the specific roles of these CBX proteins in development and disease, inspired medicinal chemists to develop compounds that disrupt this interaction. Work from the Frye lab and others have provided a catalog of compounds that target CBX4, CBX7 or CBX8. UNC3866 is a potent antagonist of CBX4 and CBX7, although it possesses affinity for other Polycomb CBX proteins and the CDY family of chromodomains (Stuckey et al., 2016). ChIP-seq studies in ESC showed that UNC3866 has a minor effect on cPRC1 stability on chromatin, yet its analog UNC4976 significantly affects cPRC1 recruitment to chromatin without affecting PRC2 activity (Lamb et al., 2019). UNC4976 is more specific towards CBX7 than UNC3866 and has a unique mechanism of action as a positive allosteric modulator of DNA and the ncRNA ANRIL binding to CBX7 (Lamb et al., 2019). This mechanism is not fully understood. To date, two specific CBX8 inhibitors have been discovered. SW2-110A has a 5-fold specificity for CBX8 compared to other Polycomb CBX proteins and reduces the binding of CBX8 to chromatin. It also inhibits cellular proliferation of leukemia cells expressing the MLL-AF9 translocation and alleviates the expression of HOXA9, an MLL-AF9 and CBX8 target (Wang et al., 2020). More recently, Bell and colleagues reported UNC7040, a specific CBX8 compound that blocks CBX8 and RING1B recruitment to PRC2 targets while, similarly to the UNC4976, enhancing CBX8 affinity to DNA. Nonetheless, UNC7040 results in cPRC1 displacement from H3K27me3-enriched genes resulting in reduced cell proliferation of cancer cells (Suh et al., 2022).

## Discussion

The importance of chromatin regulator dysfunction in cancer has become increasingly evident over the past decade, with PcG proteins being a prime example of this. Understanding the mechanisms through which the healthy and oncogenic PcG proteins control the epigenome is already yielding clinically important results (Italiano et al., 2018). However, many questions remain unanswered about their contribution to chromatin structure and function. Foremost of which is whether PHC1-3 or CBX2 mediated compaction directly contribute to repression, as acute cPRC1 knockout has little effect on transcription (Fursova et al., 2019). Importantly, cPRC1 is developmentally essential (Akasaka et al., 1996; Akasaka et al., 2001; Isono et al., 2005; Lau et al., 2017), so perhaps dynamic/differentiation model systems are required for fully elucidating its role in transcription. The contribution of phase separation to these developmental processes is still not fully appreciated as separation-of-function mutations of phase separation phenomena from other biochemical roles has proven difficult in the chromatin field. In fact, emerging data suggests phase separation does not contribute to transcriptional control in certain contexts (Trojanowski et al., 2022).

Whether the compaction functions of SUZ12, EZH1 and OGT are maintained, and physiologically contribute to gene silencing *in vivo* is also a burning question. Biochemical models supporting their compaction activities have largely been *in vitro* (Gambetta and Muller, 2014; Chen et al., 2020; Grau et al., 2021). Functional studies on PRC2 and PR-DUB compaction mutants may allow careful dissection of their contribution to chromatin compaction and gene repression in a biological system.

The contribution of histone modifications to transcriptional repression and chromatin structural organisation is becoming increasingly clear with improved genome editing and PROTAC methods for gene/protein perturbation (Dobrinic et al., 2021; Sankar et al., 2022). However, it is still unclear how promoter enriched H2AK119ub1 promotes repression in a PRC2 independent fashion (Tamburri et al., 2020; Dobrinic et al., 2021). These questions will be key to understanding how defects in PcG body formation in different cancer contexts contribute to pathogenesis and how we can leverage this to identify targeted therapeutics for patients through the multitude of available epigenetic targeted inhibitors.

It is clear that in development and oncogenic models that an imbalance in PcG and H3K36me2 domains can affect chromatin topology and, in turn, differentiation trajectories. Nonetheless, many questions remain over the functional output of these ‘blanket’ domains of histone modification. How do diffuse levels of H3K27me3 and H2AK119ub1 result in compaction (Donaldson-Collier et al., 2019; Conway et al., 2021) in the absence of accumulation of their readers CBX and JARID2/AEBP2 (Fischle et al., 2003; Kalb et al., 2014; Cooper et al., 2016; Kasinath et al., 2021)? Is this process regulated through some of the other reported readers for H2AK119ub1 such as SS18-SSX, DNMT3A or indeed the inhibitory sensing of H2AK119ub1 by NSD1/2/3 enzymes (McBride et al., 2020; Li et al., 2021; Gu et al., 2022)?

A curious commonality between the genes that regulate PcG domains is that they are often recurrently mutated in neurodevelopmental syndromes. Weavers (*EZH2*), Cohen-Gibson (*EED*), SOTOS (*NSD1*), Bohring-Opitz (*ASXL1*), Bainbridge-Roper (*ASXL3*) and Tatton-Brown-Raman (*DNMT3A*) syndromes all feature syndrome-defining mutations in chromatin regulators that control the PcG-H3K36me2 domain axis (Deevy and Bracken, 2019; Tamburri et al., 2022). This feature further highlights the importance of in-depth molecular characterisation of how these domains are regulated in order to provide clinically relevant information. The next-generation of genomics and molecular biology technologies such as PROTAC, micro-C and single cell -omics will facilitate a deeper molecular understanding of these pathways in order to maximise clinical benefit (Kaya-Okur et al., 2019; Wimalasena et al., 2020; Jerkovic and Cavalli, 2021).
